# A case report: Treatment of a patient with ruptured middle cerebral aneurysm in the second trimester of pregnancy

**DOI:** 10.1097/MD.0000000000039269

**Published:** 2024-08-16

**Authors:** Wei Li, Yang Liu, Fudi Chu, Mingjian Li, Jinpeng Wang

**Affiliations:** a Department of Neurosurgery, Weifang People’s Hospital, Weifang, Shandong, China; b School of clinical medicine, Shandong Second Medical University, Weifang, Shandong, China; c Sterilize the supply room, Weifang People’s Hospital, Weifang, Shandong, China.

**Keywords:** intracranial aneurysm, second trimester of pregnancy, surgical treatment

## Abstract

**Background:**

To summarize the clinical experience of intracranial aneurysm clipping in the treatment of ruptured intracranial aneurysms in the second trimester of pregnancy.

**Methods:**

A case of ruptured middle cerebral aneurysm in the second trimester of pregnancy was reported. Craniotomy and aneurysm clipping were performed at 24 weeks of pregnancy, and fetal preservation was continued after the operation.

**Results:**

The prognosis of the parturient was good and the skull was missing on the operative side. A healthy baby boy was delivered by cesarean section 2 months after the operation, and skull repair was performed 4 months after the operation. During the follow-up for 1 year, the mother and son were healthy and no obvious sequelae were found.

**Conclusion:**

Ruptured intracranial aneurysm hemorrhage in mid-pregnancy is a rare and critical case. Summarizing the corresponding clinical experience will help to have a reference plan for the next time when facing a similar situation, and it will help to treat critically ill patients. The treatment of ruptured intracranial aneurysm in mid-pregnancy requires multidisciplinary collaboration, and cranial aneurysm clamping + fertility preservation can reduce the impact of radiation on the fetus and improve the prognosis for both the mother and the fetus.

## 1. Introduction

The incidence of hemorrhagic stroke during pregnancy is 53/100,000, of which subarachnoid hemorrhage caused by ruptured aneurysm accounts for 16%,^[1]^ which is one of the important diseases causing death in pregnant women. In recent years, the incidence of subarachnoid hemorrhage during pregnancy showed an upward trend,^[2]^ which may be related to the opening of the second-child policy. It has been reported that^[3]^ conservative treatment and surgical treatment still have a high mortality in patients with ruptured intracranial aneurysms and fetuses during pregnancy. Therefore, we should attach great importance to the occurrence of subarachnoid hemorrhage during pregnancy. A patient with a ruptured middle cerebral aneurysm in the second trimester of pregnancy was treated with craniotomy aneurysm clipping and fetal preservation therapy. The postoperative prognosis was good and a healthy baby boy was born smoothly. Combined with the literature, the clinical characteristics and treatment of the patients were analyzed.

## 2. Case report

A 36-year-old female, 24 weeks pregnant, was hospitalized for “sudden severe headache for 12 hours.” The patient had no obvious cause and inducement for sudden severe headache on February 1, 2022, showing persistent bloating pain, nausea, vomiting, vomiting as stomach contents, no limb movement disorder and limb convulsions, and no incontinence. Magnetic resonance showed subarachnoid hemorrhage in the local hospital for further treatment. Emergency craniocerebral computed tomography angiography (CTA) showed a left middle cerebral aneurysm and was admitted to our department with “subarachnoid hemorrhage” in emergency. Admission physical examination: the Glasgow Coma Index (Glasgow Coma scale) score was 14 (awakening and moaning), and the Hunt-Hess grade belonged to grade II, clear mind, poor mental state, speech cut to the point, double pupils and other large equal circles, sensitive light response, neck resistance, thick breathing sound of both lungs, obeying activities of the limbs, and muscle tone was generally normal. Obstetrical examination: the uterine floor was flat and the fetal heart rate was 162/min. Craniocerebral computed tomography showed patchy and banded high-density shadow around the middle cerebral artery, lateral fissure cistern, tentorium, and adjacent sulcus on the right, especially in the left, lateral ventricle, high-density filling was seen in the third and fourth ventricles, and the midline was in the middle. Craniocerebral CTA showed a bag-like high-density shadow in the proximal segment of the left middle cerebral artery, bilateral anterior, middle, and posterior cerebral arteries were approximately symmetrical, and no obvious abnormality was found in the vertebrobasilar artery. Previous health, denied hypertension, diabetes and cardio-cerebrovascular diseases, 2 pregnancies, 1 delivery, parents are alive. Preoperative diagnosis: middle cerebral aneurysm (left), subarachnoid hemorrhage, 24 weeks of pregnancy, and hypertension (grade 2 high risk). (Prior to the writing of the case report in this article, the patient was informed of all relevant information and signed a written informed consent form agreeing to the use of his case for medical research and publication.)

Immediately after admission, the hospital organized multidisciplinary consultations (neonatal pediatrics, obstetrics, anesthesiology). The consultation opinion is that the gestational age of the fetus is still short, and the development of various organs is immature. If the pregnancy is terminated, the fetal survival rate is low, so it is recommended to continue the pregnancy. However, drugs and surgical trauma have a certain impact on fetal growth and development.

Changes of illness: patient suffered a sudden drop in consciousness during preoperative preparations, showed a shallow coma, no response to shouting, double pupils with equal size, 3mm in diameter, irregular left pupil, slow light response, neck resistance, limb tingling, and flexion. Heart rate 120 beats/min, blood pressure 145/80 mm Hg. Do not rule out the possibility of secondary ruptured intracranial aneurysm bleeding, immediately intravenous infusion of 20% mannitol 250 mL, do not rule out the possibility of cerebral vasospasm caused by subarachnoid hemorrhage, continue to pump nimodone, maintain the patient’s blood pressure stable, emergency surgical treatment.

**Figure 1.**
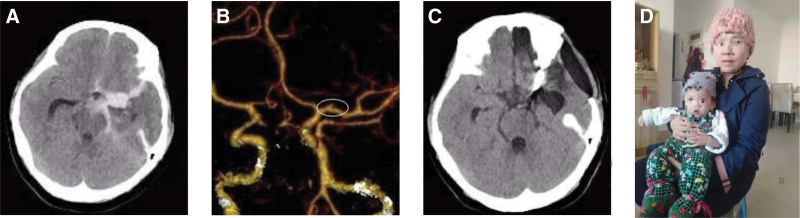
The second trimester of pregnancy complicated with a ruptured left middle cerebral aneurysm. (A) Preoperative brain CT showed patchy and banded high-density shadow around the middle cerebral artery, lateral fissure cistern, tentorium of cerebellum, and adjacent sulcus on the right, and high-density filling in the left lateral ventricle, third ventricle, and fourth ventricle. (B) Craniocerebral CTA showed a bag-like high-density shadow in the proximal segment of the left middle cerebral artery; bilateral anterior, middle, and posterior cerebral arteries were roughly symmetrical; and no obvious abnormality was found in the vertebrobasilar artery. (C) Brain CT reexamination after operation. (D) Postoperative follow-up showed that the mother and child were healthy. CT = computed tomography, CTA = computed tomography angiography.

The course of operation: craniotomy and clipping of left middle cerebral aneurysm under endotracheal intubation and general anesthesia. During the operation, the middle cerebral aneurysm was carefully separated and detected, the top of the aneurysm pointed posterior and upward, the size of the aneurysm was about 3 mm × 4 mm, the tumor body was irregular with daughter tumor, the tumor wall was thin, one aneurysm clip clipped the aneurysm neck perfectly, the parent artery was unobstructed, the brain tissue swelled obviously, and the pulsation was weak, so it was decided to remove the bone flap (Fig. 1) (patient has provided informed consent for the publication of the case).

Postoperative follow-up: the patient gave birth to a healthy baby boy 2 months after the operation, and cranial repair was performed 4 months after the operation, with a Modified Rankin scale score of 0. The prognosis was good, and the patient was dynamically followed up for 1 year after the operation, with no obvious sequelae.

## 3. Discussion

The incidence of ruptured intracranial aneurysms during pregnancy is only 0.05–1.4%,^[4]^ but its fatality rate is 50%,^[5]^ accounting for the third largest number of maternal deaths due to nonobstetric causes,^[6]^ mostly in the third trimester of pregnancy.^[7]^ The main causes of the ruptured aneurysm during pregnancy are as follows: the cardiac output of pregnant women increases gradually, and the blood volume and blood pressure increase accordingly,^[8]^ resulting in hemodynamic changes; the changes of many hormones during pregnancy. Some hormones have effects on the structure of the blood vessel wall.^[9]^ Hypertension is an independent risk factor for ruptured intracranial aneurysms.

The clinical symptoms of ruptured intracranial aneurysms during pregnancy are not significantly specific, of which about 80% are characterized by severe headache and about 20% are accompanied by limb convulsions, which need to be distinguished from nonaneurysmal subarachnoid hemorrhage and other idiopathic obstetrical diseases. Among them, the common diseases of nonaneurysmal subarachnoid hemorrhage are thrombocytopenia, hypertension, venous sinus thrombosis, coagulation disorders, and so on, accounting for about 60%^[6]^ of subarachnoid hemorrhage during pregnancy. Obstetrical idiopathic diseases include eclampsia and amniotic fluid embolism, which are often accompanied by typical hypertension and convulsions. Amniotic fluid embolism is characterized by sudden dyspnea and convulsions. All the above diseases can be distinguished by craniocerebral computed tomography and CTA, and the diagnosis can be confirmed by improving the whole cerebral angiography if necessary.

It has been confirmed that active surgical treatment of ruptured intracranial aneurysms during pregnancy can significantly improve the prognosis of parturients and fetuses.^[3]^ Surgical treatment options include craniotomy aneurysm clipping and transcatheter interventional embolization of intracranial aneurysms. The choice of surgical scheme, the timing of surgical intervention, and the mode of delivery vary according to different gestational weeks. The patient is in the second trimester of pregnancy, comprehensively considering the situation of the pregnant woman and the fetus. The patient was in the middle stage of pregnancy, and considering the situation of the pregnant woman and the fetus, although there have been many studies confirming^[10–12]^ the successful cases of successful delivery of fetuses by measures such as protecting the pregnant woman’s abdomen with a lead garment and reducing the time of exposure, only short-term follow-up is available, and the lack of long-term follow-up of the deformity rate and the therapeutic efficacy is not exact, so the decision was made to choose open aneurysm clipping + fertility preservation treatment. The advantages of this surgical scheme are as follows: to reduce the impact of interventional therapy on the fetus. Radiation in the middle and third trimester of pregnancy mainly affects the growth of fetal neurons, leading to growth patients, microcephaly, etc.^[9]^ It avoids the possibility of long-term oral administration of antiplatelet drugs such as aspirin after stent-assisted interventional embolization and reduces the risk of massive bleeding during cesarean section or delivery caused by oral aspirin. In cases of ruptured intracranial aneurysms in pregnant women in early pregnancy, neurosurgical treatment should be based on the principles of active resuscitation of the pregnant woman, and the continuation of pregnancy should be decided according to the condition of the pregnant woman and the condition of the fetus after the condition is stabilized. For pregnant women in the third trimester of pregnancy (especially after 34 weeks), termination of pregnancy can increase the probability of neonatal delivery because the fetal lungs have matured. Interventional embolization or clipping of intracranial aneurysms is feasible for intracranial aneurysms.^[13]^ As holding breath during delivery will increase the risk of secondary rupture of the aneurysm, it is recommended to perform a cesarean section under general anesthesia. If conditions permit, cesarean section and intracranial aneurysm can also be performed at the same time. The above conditions are only for Hunt-Hess grade I–III, and the vital signs are relatively stable; if Hunt-Hess grade IV and V, the vital signs of pregnant women are unstable, mainly to remove intracerebral hematoma, clip aneurysm and save the life of pregnant women, and detect fetal heart rate at the same time. If the fetal lung is mature and respiratory distress occurs during the operation, a cesarean section will be performed immediately.^[14]^ The prognosis of puerpera and fetus is not only related to the choice of operation scheme but also the perioperative nursing and the application of anesthetics: to strictly control the blood pressure before operation to stabilize the mood of pregnant women and to avoid the secondary rupture of aneurysm; anesthetics and analgesics can easily enter the fetus through the placental barrier. All of them to the central system have an inhibitory effect, and excessive anesthetic drugs will inhibit the breathing of pregnant women and lead to fetal respiratory distress.

In summary, the treatment of ruptured intracranial aneurysm in pregnancy requires multidisciplinary collaboration, and different surgical methods and delivery methods are selected according to the condition of the pregnant woman and the gestational week of the fetus. The choice of cranial aneurysm clamping + fertility preservation in mid-pregnancy with ruptured intracranial aneurysm hemorrhage can reduce the impact of radiation on the fetus and improve the prognosis of both the mother and the fetus. At the same time, the perioperative care of pregnant women and the use of anesthesia drugs are also crucial. The study has some limitations, the number of cases is too small, which may be due to chance, and the follow-up period is only 1 year, which still needs to focus on the health status of the mother and child for a longer period of time, and the methodology requires a certain level of sophistication for the relevant departments in the hospital.

## Author contributions

**Writing – original draft:** Wei LI, yang Liu, Fudi Chu

**Writing – review & editing:** Fudi Chu, Mingjian Li, Jinpeng Wang

## References

[1] Liu XJ Wang S Zhao YL. A single-center study of hemorrhagic stroke caused by cerebrovascular disease during pregnancy and puerperium in China. Int J Gyneco Obstet. 2011;113:82–3. 10.1016/j.ijgo.2010.11.010 21334622

[2] Kuklina EV Tong X Bansil P George MG Callaghan WM. Trends in pregnancy hospitalizations that included a stroke in the United States from 1994 to 2007: reasons for concern? Stroke. 2011;42:2564–70. 21799174 10.1161/STROKEAHA.110.610592

[3] Dias MS Sekhar LN. Intracranial hemorrhage from aneurysms and arteriovenous malformations during pregnancy and the puerperium. Neurosurgery. 1990;27:855–65; discussion 865. 2274125 10.1097/00006123-199012000-00001

[4] Kim YW Neal D Hoh BL. Cerebral aneurysms in pregnancy and delivery: pregnancy and delivery do not increase the risk of aneurysm rupture. Neurosurgery. 2013;72:143–9; discussion 150. 23147786 10.1227/NEU.0b013e3182796af9

[5] Lovelock CE Rinkel G Rothwell PM. Time trends in outcome of subarachnoid hemorrhage: population-based study and systematic review. Neurology. 2010;74:1494–501. 20375310 10.1212/WNL.0b013e3181dd42b3 PMC2875923

[6] Bateman BT Olbrecht VA Berman MF Minehart RD Schwamm LH Leffert LR. Peripartum subarachnoid hemorrhage: nationwide date and institutional experience. Anesthesiology. 2012;116:324–33. 22166951 10.1097/ALN.0b013e3182410b22

[7] Desai M Wali AR Birk HS Santiago-Dieppa DR Khalessi AA. Role of pregnancy and female sex steroids on aneurysm formation, growth, and rupture: a systematic review of the literature. Neurosurg Focus. 2019;47:E8. 10.3171/2019.4.FOCUS19228 31261131

[8] Lamy C Hamon JB Coste J Mas JL; for the French Study Group on Stroke in Pregnancy. Ischaemic stroke in young women: risk of recurrence during subsequent pregnancies. French Study Group on Stroke and Pregnancies. Neurology. 2000;55:269–74. 10908903 10.1212/wnl.55.2.269

[9] Wiebers D. Unruptured intracranial aneurysms: natural history, clinical outcome, and risks of surgical and endovascular treatment. Lancet. 2003;362:103–10. 12867109 10.1016/s0140-6736(03)13860-3

[10] Piotin M Filho CS Kothimbakam R. Endovascular treatment of acutely ruptured intracranial aneurysms in pregnancy. Am J Obstet Gynecol. 2001;185:1261–2. 11717668 10.1067/mob.2001.115861

[11] Pumar J Pardo M Carreira J. Endovascular treatment of an acutely ruptured intracranial aneurysm in pregnancy: report of eight cases. Emerg Radiol. 2010;17:205–7. 19921289 10.1007/s10140-009-0848-0

[12] Kizilkilic O Albayram S Adaletli I. Endovascular treatment of ruptured intracranial aneurysms during pregnancy: report of three cases. Arch Gynecol Obstet. 2003;268:325–8. 14504879 10.1007/s00404-002-0384-6

[13] Barbarite E Hussain S Dellarole A Elhammady MS Peterson E. The management of intracranial aneurysms during pregnancy: a systematic review. Turk Neurosurg. 2016;26:465–74. 27400091 10.5137/1019-5149.JTN.15773-15.0

[14] Kataoka H Miyoshi T Neki R. Subarachnoid hemorrhage form intracranial aneurysms during pregnancy and the puerperium. Neurol Med Chir (Tokyo). 2013;53:549–54. 23979051 10.2176/nmc.53.549

